# Genetically Engineered-MSC Therapies for Non-unions, Delayed Unions and Critical-size Bone Defects

**DOI:** 10.3390/ijms20143430

**Published:** 2019-07-12

**Authors:** Jaime Freitas, Susana Gomes Santos, Raquel Madeira Gonçalves, José Henrique Teixeira, Mário Adolfo Barbosa, Maria Inês Almeida

**Affiliations:** 1 i3S—Instituto de Investigação e Inovação em Saúde, University of Porto, 4200-465 Porto, Portugal; 2 INEB—Instituto de Engenharia Biomédica, University of Porto, 4200-465 Porto, Portugal; 3 ICBAS—Instituto de Ciências Biomédicas Abel Salazar, University of Porto, 4200-465 Porto, Portugal

**Keywords:** regeneration, osteogenic differentiation, immunomodulation, bone repair

## Abstract

The normal bone regeneration process is a complex and coordinated series of events involving different cell types and molecules. However, this process is impaired in critical-size/large bone defects, with non-unions or delayed unions remaining a major clinical problem. Novel strategies are needed to aid the current therapeutic approaches. Mesenchymal stem/stromal cells (MSCs) are able to promote bone regeneration. Their beneficial effects can be improved by modulating the expression levels of specific genes with the purpose of stimulating MSC proliferation, osteogenic differentiation or their immunomodulatory capacity. In this context, the genetic engineering of MSCs is expected to further enhance their pro-regenerative properties and accelerate bone healing. Herein, we review the most promising molecular candidates (protein-coding and non-coding transcripts) and discuss the different methodologies to engineer and deliver MSCs, mainly focusing on in vivo animal studies. Considering the potential of the MSC secretome for bone repair, this topic has also been addressed. Furthermore, the promising results of clinical studies using MSC for bone regeneration are discussed. Finally, we debate the advantages and limitations of using MSCs, or genetically-engineered MSCs, and their potential as promoters of bone fracture regeneration/repair.

## 1. Introduction

Bone repair and regeneration is a complex process that involves a well-orchestrated series of events engaging several cell types and molecular signaling pathways in a precise spatiotemporal sequence [1]. Among the different cell types involved in this process, mesenchymal stem/stromal cells (MSCs) play a pivotal role in bone fracture repair [1]. These cells are multipotent (capable of self-renewal and differentiation into distinct lineages—osteoblasts, adipocytes and chondrocytes), and usually characterized as expressing the surface antigens CD73, CD90 and CD105 (positive markers), and not expressing antigens characteristic particularly of hematopoietic lineage, like CD34, CD35, CD19, CD14 and HLA-DR (negative markers), and having the capacity to adhere to plastic in vitro [2]. Upon bone fracture, MSCs migrate to the injury site and can differentiate into bone-forming osteoblasts, a process called osteogenic differentiation, ultimately maturing into osteocytes that become entrapped in the bone matrix. In some cases, MSCs can also differentiate into cartilage forming chondrocytes that serve as a cartilaginous template for the formation of new bone, in a process of endochondral ossification [3]. Another important feature widely reported for MSCs is their capacity to modulate the immune response and inflammation upon injury, and the production of survival, proliferation, angiogenic and osteoinductive factors [4]. Due to their exceptional characteristics, MSCs have been extensively studied in several cell-based therapies, including cell transplantation, tissue engineering, gene therapy, and immunotherapy [4].

Due to an increased life expectancy, the number of individuals that suffer bone fractures worldwide is expected to double by 2020 [5,6]. The bone regeneration process can be impaired as a consequence of the size of the injury. High-energy traumas, tumors resections, prosthetic and revision surgeries, bone diseases (including osteoporosis) and infections can cause large/critical size bone defects that may lead to non-unions or delayed unions [5,6]. Presently, non-unions and delayed unions are estimated to account for approximately 5–10% of bone fractures, but the prevalence depends on several criteria such as the anatomic region [7]. These non-healing fractures have a high financial impact for health systems, and also present indirect economic costs associated with loss of productivity and absence from work [6]. Therefore, new strategies are needed to solve these clinical problems and accelerate recovery time for patients with difficult-to-heal bone fractures.

Currently, the gold standard to treat non-union and delayed union fractures is still the use of autologous bone grafts. However, the limited availability, the need for a second surgery to harvest bone and other complications emphasize the need for the development of alternative clinical strategies. Recently, clinical treatment approaches for non-unions were reviewed by Schulundt et al. [8]. Cell-based therapies to treat large bone defects have mainly focused on the use of MSCs, mostly because they can be easily obtained from different tissues, and have capacity to differentiate into relevant cell lineages, like osteogenic and chondrogenic. The most commonly used sources of MSC are bone marrow (BM), adipose tissue, umbilical cord, and placenta tissue [9]. Another source for collection of cells with MSC-like characteristics are human oral tissues, including dental pulp stem cells (DPSCs), periodontal ligament stem cells (PDLSCs), apical papilla stem cells (SCAPs), dental follicle stem cells (DFSCs), stem cells from exfoliated deciduous teeth (SHED) and gingival tissue stem cells (GMSCs) [10,11,12].

MSC-based therapies have shown to be a viable alternative with promising advantages in repairing the structure and function of the damaged bone, which is particularly relevant for improving the regeneration of large fractures/non-unions [8]. Reports comparing MSCs from different origins seeded on biologically active scaffolds indicate that human BM-derived MSCs are more efficient than embryonic stem cell-derived MSCs [13], adipose tissue and umbilical cord-derived MSCs [14], with BM-MSC promoting further osteogenesis, while umbilical cord-MSCs promoted angiogenesis [14]. Comparing MSCs derived from induced pluripotent stem (iPS) cells (from peripheral blood or umbilical cord), and from human embryonic stem cells, similar osteogenic capacity to BM-derived MSCs was found [15]. However, the authors found differences in angiogenic potential, which are reflected in their ability to repair rat femur non-union fractures. BM and umbilical cord-iPS derived cells were more angiogenic and more efficient in bone repair, producing more vascular endothelial growth factor (VEGF) or basic fibroblast growth factor (FGFb) than cells from other origins [15]. When comparing MSCs with other cell populations, human adipose-derived stem cells implanted in immunodeficient rats improved healing, when compared to implanting fibroblasts or with PBS control, due to increased bone morphogenetic protein-2 (BMP-2) and VEGF expression in peri-fracture tissue [16].

Typically, MSC-based therapies involve either the isolation of cells (with or without in vitro expansion) and direct transplantation to the damaged tissue for in vivo differentiation, or cells are genetically engineered to express specific genes that improve their features, including lineage-specific differentiation, prior to implantation [17]. This will be explored in the next chapters, with a highlight into animal in vivo or clinical studies.

## 2. Molecular Candidates and Common Methods to Genetically-Engineer MSC to Promote Bone Repair

### 2.1. Genes of Interest to Promote Bone Repair in vivo: Promising Candidates

Most genetically modified MSC-based studies involve the osteoinductive bone morphogenetic proteins (BMPs), which are members of the transforming growth factor-beta (TGF-β) family and are known to play an important role in the regulation of bone induction, maintenance and repair [18]. Other genes of interest include transcription factors essential for osteoblast differentiation (the core binding factor α1 Cbfa1 [19] and Osterix/Osx [20]). This has been shown by Zheng and colleagues by transplanting a type I collagen scaffold seeded with MSCs transduced with Cbfa1 into a 5 mm diameter critical-sized skull defect in mice, resulting in an 85% osseous closure in four weeks compared to control [19]. In a similar study, Tu et al. showed that the local implantation of Osx-transduced BM-MSCs, with type I collagen sponges as a carrier, resulted in a five times improvement of calvarial bone defect healing in adult mice, in comparison with mice transplanted with empty vector transduced BM-MSCs [20].

Factors enhancing angiogenesis, such as the VEGF, have also been used in conjunction with BMP-2 to stimulate angiogenesis and bone formation. Specifically, VEGF and BMP-2 induced the homing of tail vein injected BM-MSCs to the implantation site of a silk scaffold in nude mice [21]. Remarkably, the implantation of BM-MSCs engineered by baculovirus to express BMP-2 and VEGF into critical-sized femoral segmental defects in rabbits improved the repair of the fractures and the quality of the regenerated bone [22]. Additionally, the use of the BMP antagonist Noggin was shown by Pen et al. to be an interesting approach to regulate bone formation in vivo [23]. Co-implantation of MSCs transduced with BMP-4 and Noggin into critical-sized calvarial defects in mice prevented bone overgrowth and led to the regeneration of bone that more closely resembled the normal bone. Exploiting a different approach, Simonsen et al. were able to extend the lifespan of MSCs for tissue engineering applications [24]. The authors stably transduced MSCs with a vector containing the catalytic subunit of human telomerase (hTERT) increasing the mean telomere length, while maintaining the expression of osteoblastic markers and their differentiation potential. Moreover, subcutaneous implants of these transduced MSC-TERT cells in immunodeficient mice resulted in improved bone formation, suggesting that TERT expression in MSCs prevents the loss of osteoblast functions due to senescence [24].

Considering that epigenetic regulation plays an important role in the induction of MSC differentiation, regulators of DNA methylation and histone modifications lead to changes in the MSC profile and, thus have potential as tools to engineer MSCs [25]. For instance, Paino et al. showed that HDAC2 silencing by RNA interference increased the expression of osteopontin (OPN) and bone sialoprotein (BSP), while downregulating OC [26]. Following this work, La Noce et al. showed that the OC reduction was mediated by glucocorticoid receptor, which translocates into the nucleus in the absence of HDAC2 and represses osteocalcin (OC) transcript [27]. Also, authors showed that treatment of human dental pulps with valproic acid, a HDAC inhibitor that decreases HDAC2, led to bone tissue formation with a lamellar compact bone tissue structure, when cells were implanted subcutaneously using immunodeficient mice [27]. Therefore, some epigenetic regulators can be interesting candidates to induce bone formation.

Besides MSC genetic transformation to express particular protein coding genes, in recent years, the use of non-coding RNAs (ncRNAs) that have an impact in different aspects of MSC biology, has been gathering exponential interest. Among these, microRNAs (miRNAs) have been the most extensively studied class due to their potential to directly regulate the migration of MSCs [28], their differentiation lineage [29], or their immunomodulatory capacity [30]. Specifically, miRNAs are a class of small ncRNAs that act as post-transcriptional regulators of gene expression, generally by binding to 3’ UTR of the target messenger RNA (mRNA) [29,31], and that have been associated with the regulation of several cell biology processes, including apoptosis, proliferation, migration, differentiation and apoptosis [32]. Thus, miRNAs mimics, anti-miRNA/antagomiRs or plasmids to overexpress or inhibit miRNA levels have been used to modulated MSC and induce bone formation in in vivo studies [33].

Reports show that overexpressing miR-21 accelerates rats fracture healing in a closed femur fracture model [34]. Interestingly, a recent study profiling the circulating miRnome in a rat critical size bone injury model found a temporal regulation of miR-21, with an upregulation at 14 days, when compared to 3 days, post-injury [35]. Also, the essential role of miR-21 in the migration and osteogenic differentiation of BM-MSCs was recently demonstrated, by implanting miR-21-modified BM-MSCs/β-TCP scaffold into critical-size defects in rats and dogs, which resulted in a significantly higher amount of new bone formation [36]. These in vivo results are in agreement with in vitro data that showed this miRNA has an anti-apoptosis role in MSCs and is able to promote cell migration via the regulation of the PTEN/PI3K/Akt pathway [37].

In a femur fracture mouse model, locally administered lentiviral transduced MSC to overexpress miR-218 were able to promote bone healing and improved the quality of new callus after local administration [38]. For several miRNAs, their bone pro-regenerative effect is induced when miRNA levels are inhibited, as it is the case of miR-221. Specifically, PCL/HA nanofibers seeded with rat MSCs transfected with anti-miR-221 could enhance bone healing of the rat skull [39]. The authors compared non-transfected MSCs versus anti-miR-221-transfected MSCs, both seeded on PCL/HA scaffold, and used as controls the scaffold without cells or the empty bone defect [39]. Interestingly, the results showed that at 8 weeks after the surgery to induce the bone defect, the combination of MSCs (either non-transfected or transfected with anti-miR-221) with the scaffold showed increased hard tissue formation compared to the control groups [39]. However, at an earlier time point (4 weeks post-surgery) only the anti-miR-221-transfected MSCs, and not the non-transfected MSCs, showed a partial bone healing, suggesting the anti-miR-221 could have a beneficial effect [39].

Also, Yoshizuca et al. showed that inhibition of miR-222 accelerates osteogenic differentiation, while overexpressing this miRNA causes the contrary effect [40]. Thus, miR-222 knockdown led to bone union in a rat femoral transverse fracture model [40]. Also, baculovirus engineered MSC to express miRNAs, like miR-148b and miR-196a, have been reported to improve bone repair [41], while in another study the same system was used to express sponges (anti-miRNA) for miR-214 to improve bone repair in the ovariectomized model of osteoporosis ^42^. In both studies, co-expression of BMP-2 improved bone healing [41,42].

Furthermore, Xie et al. demonstrated the in vivo effect of adipose-derived MSCs after lentiviral transduction with miR-135, a pro-osteogenic miRNA that directly targets HOXA2 and is able to induce expression of osteogenic markers such as Runt-related transcription factor 2 (Runx2), OC, BSP and OPN [43]. The combination of miR-135 overexpressing rat adipose-derived MSCs with poly(sebacoyl diglyceride) scaffold to repair a critical-sized calvarial defects significantly promoted new bone formation [43]. A similar effect has been observed in the same animal model using miR-31-transduced adipose tissue derived stem cells with β-tricalcium phosphate (β-TCP) scaffolds [44].

Finally, another class of ncRNAs, called “long non-coding RNAs”, have recently been explored as mediators of bone repair and modulation of their levels in MSCs has been tested. For detailed revision on this topic please see [45,46,47].

### 2.2. MSCs Engineering Strategies to Express/Inhibit Genes of Interest

Most often, genetically modified MSCs are generated by exploiting viral-based gene-delivery systems. However, these systems raise safety concerns regarding their use in humans due to issues involving immunogenicity and stimulation of immune-modulatory responses. Hence, the success of therapies based on genetically modified MSCs is largely dependent on the development of gene-delivery systems that confer high levels of expression with minimal toxicity and without the ability to stimulate any specific immune response *in vivo*. Alternatives to viral delivery systems with potential to be used for gene therapy in different clinical settings are usually based on direct gene transfer of naked plasmid DNA via physical and chemical methods [48], which include gene transfer via gene guns or biolistic particle delivery systems [49], electroporation [50], liposomes and polymer-based systems [51]. Li et al. successfully improved bone callus formation in New Zealand white rabbits with critical-sized segmental bone defects by bombarding the fracture site with gold-coated plasmid DNA encoding BMP-2 with a helium-driven gene gun [49]. Ferreira et al. have also shown the feasibility of using electroporation for efficient gene transfer into rat BM-MSCs while preserving cell viability and multipotency [52]. A study by Park and colleagues compared the use of adenoviral vector to liposomes and concluded that both liposome-mediated and adenoviral BMP-2 gene transfer to primary BM-MSCs are suitable methods to heal critical-size bone defects in rats, with the liposomes having the advantage of being easy to prepare and less prone to elicit a immune response [53]. Also, using BMP2-modified MSCs, Bluim et al. compared in vivo bone formation in a rat orthotopic critical-size defect, following the delivery of MSCs expressing hBMP-2 through 3 methods, namely adenoviral, retroviral, and cationic lipid vectors [54]. Results showed that adenovirus BMP-2 modified MSCs had an increased bone formation compared with the other tested methodologies [54].

MSCs can also be genetically modified using a gene-activated matrix (GAM). GAM consists of collagenous scaffold saturated with the plasmid DNA that encodes for the osteoinducible factor. GAMs are usually inserted locally into the bone fracture site, allowing for the transfection of the invading MSCs and thus expression and secretion of the osteoinducible factor. This “cell free” gene-activated scaffold platforms have the advantage of locally transfecting the host cells that infiltrate and proliferate on the scaffold implanted at the defect site, without the need of ex vivo or in vitro transfection [55]. Umebayashi et al. have successfully used this method in the transplantation of GAMs for the expression of BMP-4 and Runx2 in the cranial bone surface under the periosteum of F344 rats, resulting in a GAM dose-dependent bone induction [56]. Another study has shown an accelerating bone repair in rat critical-size calvarial defect when nanoparticles containing both BMP-2 and VEGF plasmids were incorporated into bone mimicking collagen hydroxyapatite scaffolds [57]. This is particularly interesting considering the simultaneous induction of a pro-osteogenic and a pro-angiogenic gene [57].

In vivo electroporation has also shown the potential to genetically modify MSCs to effectively induce bone formation. This has been shown in a study by Kawai and colleagues, in which simultaneous transfer of BMP-2 and BMP-7 by intramuscular DNA injection and transcutaneous electroporation resulted in better-defined opacities in the muscles of rats that received the treatment. Further histological examination showed advanced ossification compared to rats electroporated either gene alone [58].

Additionally, genome editing techniques like CRISPR/Cas9 hold great potential in the production of genetically modified MSCs. Recently, by repurposing the technique for CRISPR interference (CRISPRi), by using a catalytically inactive Cas9 (dCas9) which orchestrates with sgRNA to sterically block the transcription of target genes, Truong et al. were able to simultaneously activate Sox9 and repress PPAR-γ in rat BM-MSCs [59]. Importantly, implantation of these CRISPRai-engineered BM-MSCs seeded into gelatin scaffolds improved the healing of critical-size calvarial bone defects in rats [59].

### 2.3. Tracking MSC for in vivo Studies

Transplanted MSCs *in vivo*, either non-genetically modified MSC or engineered-MSC, is generally studied through histology methods, which enables the collection of data for a single time point per experimental animal, leading to the use of a high number of animals, which does not comply with the most recent ethical recommendations. In contrast, live cell imaging allows to track in real-time the migration dynamics of grafted MSCs. This is usually performed by labelling the MSCs with reporter genes via viral or non-viral vectors [60,61]. In a study designed that evaluated the in vivo effect of Osx-overexpressing BM-MSCs, the authors used a green fluorescent protein (GFP) vector to determine the source and fate of the regenerative cells in the implantation site using immunohistochemistry for GFP [20]. Similarly, Zhang and colleagues labelled with lenti-GFP autogenic rabbit BM-MSCs, enabling cell tracking upon their reinjection via the ear vein, to determine whether VEGF and BMP-2 improve the homing and differentiation of these cells into the skull defect site [21]. Additionally, bioluminescence imaging can also be used to analyze the bone repair potential of different cells types and materials. Dégano et al. used luciferase, a commonly used reporter gene for in vitro studies, to effectively monitor and quantify the proliferation of virally transduced luciferase-labelled MSCs seeded in osteoconductive hydrogel scaffolds during extended periods of time (90 days) [60]. Nonetheless, the use of viral methods and the need for genomic manipulation raises safety issues. To overcome these issues molecular probes and fluorescent nanoparticles were developed. Zong et al. reported the use of the fluorescent probe carbocyanine CM-Dil to label the cell membranes of hMSCs and osteoblast-like cells in scaffolds grafted into critical-size calvarial defects in rats [62]. Ten weeks after transplantation, the graft area was harvested for cell tracing analysis via fluorescein microscopy [62]. Quantum dots have also been used in stem cell research for in vivo bioimaging, as seen in the work of Dupont and colleagues that implanted scaffolds containing fluorescent quantum dots (QDs)-labeled hMSCs into critically sized femoral defects in rats [63]. Scans revealed a clear fluorescent signal at the defect site in the right hindlimb, which decreased rapidly but remained above background level [63]. Recently, Lee et al. reported the use of fluorescent silica nanoparticles to label human MSCs to be injected into the bone marrow cavity of nude rats through osteochondral defects created in the distal femur [64]. This allowed for the detection of the time-dependent migration of MSCs within the bone marrow to the defect for 21 days [64].

## 3. MSC Delivery to Promote Bone Regeneration

### 3.1. Local Delivery of MSC: The Combined Effect with Biomaterial Scaffolds

Bone grafts are still the most common procedure to treat skeletal fractures. Of these, autografts are by far the most frequent, though it can lead to several adverse effects such as pain, infection and donor-site morbidity [65]. Allografts, can be used as an alternative, but also present limitations such as the risk of contamination with infectious agents or immune tissue rejection [66]. Due to the drawbacks associated with both approaches, the field of bone regenerative medicine has exploited new biomaterials and scaffolds in association with bone progenitor cells and growth factors to stimulate bone repair and regeneration. The use of scaffolds supports the local delivery of MSCs into the bone defects, reducing the risk of ectopic bone formation. In this context, biomaterials to which MSCs can easily adhere should be considered. Ideally, a scaffold should also enhance MSC viability and promote MSC osteogenic differentiation. The characteristics, advantages and limitations of bone tissue engineering scaffolds have been extensively revised by De Witte et al. [67]. In this chapter, we provide examples of studies that used scaffolds to seed MSC in bone defect animal models.

Ceramic biomaterials are generally composed of calcium phosphate (CaP), having the advantage of being biocompatible, osteoconductive and resistant to compression and corrosion. The most common CaP-based scaffolds are hydroxyapatite (HA), beta-tricalcium phosphate (β-TCP) and a combination of these, the biphasic calcium phosphate (BCP). Hirasawa et al. showed that patients that underwent lumbar interbody fusions treated with a CaP cement achieved a fusion rate similar to the use of autografts [68]. CaP-based biomaterials are nonetheless highly brittle and present low osteoinductivity, the latter shortcoming can be circumvented with the combined use of BMPs resulting in a scaffold with greater osteoinductive capacity [69]. Additionally, MSCs can also be combined with BMP-loaded CaP-based biomaterials to enhance bone repair. This has been shown by adding rhBMP-2 to silica-coated calcium hydroxyapatite (HASi) scaffolds seeded with rabbit BMSCs to promote bone healing in large segmental bone defects (30 mm) in rabbits [70]. Moreover, using BMSCs isolated from patients and seeded onto porous hydroxyapatite, Marcacci et al. were able to treat four patients with large bone diaphysis defects [71].

Polymer biomaterials can either be of natural or synthetic origin, with each type providing unique properties. Natural polymers offer the ability to mimic the structure and biochemical properties of the natural bone matrix, with the disadvantage of having poor thermal stability, processability and degradation control [72]. These include, for instance, collagen, the most abundant protein in the bone matrix [73], which in combination rBM-MSCs has been shown to promote bone repair in a rat calvaria critical-size defect model comparable to the same matrix material combined with BMP-2 [74]. Chitosan, a natural linear polysaccharide is able to enhance the production of cytokines and growth factors necessary for bone repair [75]. The combination of chitosan-PBS scaffolds with human BM-MSCs implanted into critical-sized cranial defects in nude mice resulted in enhanced integration with the surrounding tissue and significant bone formation [76]. Alternatively, demineralized bone matrices (DBM), a natural bone substitute derived from bones lacking mineral components, can also be used in the treatment of non-union fractures. Desai et al. have shown that a percutaneous bone marrow aspirate concentrate (BMAC) injection combined with DBM is effective in treating tibial non-unions with fracture gaps less than 5 mm [77].

Synthetic polymers generally offer a higher degree of control over the physiochemical characteristics of the scaffolds (e.g., pore size, solubility, biocompatibility and immune response). These include the aliphatic polyesters poly(lactic-acid) (PLA), poly(glycolic-acid) (PGA), poly(lactic-coglicolide) (PLGA), poly(caprolactone) (PCL) and their copolymers which are most frequently used in bone tissue engineering [78]. PLA-based biomaterials have shown to support bone healing through the adhesion and growth of human osteoprogenitor cells [79]. A complete reconstruction of the cranial vault was observed in rats with critical-size craniotomy defects grafted with scaffolds made of PLA/ceramic constructs [80]. The use of PGA biomaterials is quite limiting due to the low osteoconductivity and compressive strength, nonetheless PLGA copolymers with different ratios of PLA and PGA present a much higher osteoconductivity and controllable degradation times [81]. By implanting a PLGA scaffold seeded with MSCs pre-differentiated in vitro into cartilage-forming chondrocytes, Harada et al. were able to heal both critical-sized (5 mm) and massive (15 mm) full thickness femur defects in rats [82].

The FDA approved PCL is by far the most studied polymer, due to its high permeability, thermal stability and highly biocompatibility [83]. Importantly, synthetic polymers, in general, also present disadvantages, mainly because their degradation products can change the local pH resulting in cell growth inhibition and inflammation [84,85]. Studies have shown that the acidic degradation products can be neutralized through the addition of alkaline materials [86] or construction of composites with ceramic biomaterials or other polymers [87,88]. In 2011, Bassi et al. have shown the healing potential of a bone graft substitute composed of PCL and poly (vinyl phosphonic acid-*co*-acrylic acid) in critical-size defects in mouse calvaria [89]. Moreover, the combination of genetically engineered rat bone marrow derived MSCs overexpressing Runx2 with fused deposition-modeled scaffolds composed of biodegradable PCL resulted in a two-fold increase in bone formation in critical-size craniotomy defects in rats [90].

Derived from a combination of polymers and ceramics, composite biomaterials were developed as to integrate the advantages of both classes and reduce their disadvantages [91]. Composite biomaterials of combined hydroxyapatite and collagen have shown to promote osteogenic differentiation in MSCs derived from human adipose tissue through the induction of osteogenic genes resulting in increased cell viability and matrix mineralization [92]. Similarly, a collagen-magnesium-enriched hydroxyapatite (MHA) scaffold was ectopically implanted into mice showing an improved recruiting host cells that invade the scaffold and promote bone augmentation [93]. Using a mouse pre-osteoblastic (MC3T3-E1) cell line Wang et al. reported a higher attachment and proliferation of pre-osteoblasts using a composite scaffold of a PLA/β-TCP matrix grafted with gelatin/hydroxyapatite represents a good candidate for bone repair [94]. An increase in porcine BM-MSCs proliferation and differentiation was also shown using a scaffold composed of poly (ε-caprolactone)/tricalcium phosphate (PCL/TCP) with carbonated hydroxyapatite (CHA)-gelatin [95]. Collagen/β-TCP composites were used as bone void filler in the critical-size defects of rabbit distal femoral condyle model, showing both the resorption of the composite and bone formation in the defect without ant toxic or immune response [96].

Recent advances in nanotechnology have created the possibility to develop nanoparticles (NPs) that can also enhance scaffold properties, providing greater mechanical properties, osteointegration, osteoconduction, and osteoinduction [97,98]. Currently there are no commercially available applications of NPs for bone regeneration, although in recent years, several studies have shown the potential of the technology, particularly in the development of improved scaffolds for the repair of critical-sized bone defects. Cao and co-authors reported on the use of BMP-2 loaded nanocomposite biomaterials to efficiently obtain osteoinductivity and enhance the repair of critical-sized bone defects. In this study, a gelatin sponge (G) loaded with a 2-*N*,6*-O-*sulfated chitosan (26SCS) based nanoparticle (S-NP) encapsulating BMP-2 was used to fill critical-size defects in rabbit radius, improving vascularization during orthotopic bone formation [99]. Li and colleagues developed a scaffold also through the encapsulation of BMP-2 into bovine serum albumin (BSA) nanoparticles and the *co*-electrospinning of the BMP-2/BSA nanoparticles, dexamethasone and the poly(ε-caprolactone)-*co*-poly(ethylene glycol) (PCE) copolymer [100]. The controlled dual delivery of BMP-2 and DEX significantly improved the efficiency of bone repair in critical-size rat calvarial defects [100]. Recently, engineered extracellular vesicles (EV) have also been used to promote bone regeneration. This was the strategy followed by Pizzicannella et al. that coated hPDLSC-derived EVs with polyethylenimine (PEI) to induce intracellular release [101]. Specifically, authors showed that collagen-based scaffolds with hPDLSC and PEI-EVs increased the bone regenerative capacity in calvarial defects, by promoting the expression of osteogenic markers (RUNX-2, BMP2/4 and COL1A1) and also the activation of pro-angiogenic factors, such as VEGF and VEGF receptor 2 (VEGFR2) [101]. A different approach was employed by Qureshi and colleagues by using nanoformulated miRNA-based conjugates released in scaffolds via photoactivation. In this report, human adipose tissue-derived stem cells (ASCs) were transfected with miR-148b bound to silver nanoparticles (SNPs) through a photolabile linker and loaded to a PCL scaffold, improving the closure of a critical-size defect on the right parietal bone of male CD-1 athymic nude mice [102].

### 3.2. Systemic Delivery of MSC for Bone Repair

The systemic enhancement of bone regeneration has the potential to overcome some of the limitations of the current gold standard bone repair therapies based on local intervention. The systemic treatments offer the possibility to accelerate the overall regeneration process in skeletal disorders such as osteoporosis, enables the repair of multiple fracture sites and eliminate the need for surgery, greatly reducing the potential for postoperative complications such as infection. Moreover, homing of MSCs plays a central role in bone fractures repair, being the recruitment of MSC to the injury site mediated by several chemokines and trophic factors [103]. Dissection of the mechanisms and molecules that promote MSC homing could provide important evidences to improve bone repair following MSC systemic administration.

Systemic injection of MSCs for the treatment of skeletal disorders and bone injuries has been the focus of several studies throughout the most recent years. Granero-Molto et al. reported in a stabilized tibia fracture mouse model that transplanted allogeneic MSCs migration to the fracture site is CXC chemokine receptor type 4 (CXCR4)-dependent and these MSCs contribute to the mouse fracture repair by expressing BMP-2 at the fracture site [104]. Lien and colleagues found that MSCs overexpressing CXCR4 have a higher retention and homing in bone marrow when compared to non-modified MSCs, transplanted by intramedullary and tail vein injections, respectively, into immunocompetent mice [105]. Additionally, authors report a complete recovery of bone stiffness and strength in glucocorticoid-induced osteoporotic mice after a systemic transplantation of CXCR4 and Cbfa-1 co-transduced MSCs, which also suggests that genetically-engineered MSCs can be exploited for bone repair and regeneration [105]. The bone healing efficacy of delivering MSCs intravenously was also shown by injecting green fluorescent protein (GFP)-labelled MSCs in a mouse femoral osteotomy model [106], where a significant increase in bone volume was observed in the MSC-treated group. Using a rat closed transverse femoral fracture model, a more recent study compared the contribution to bone repair of allogeneic MSCs delivered locally or systemically via heart injection and found that either could promote fracture repair significantly, without inducing any noticeable immune response [107]. Notably, the number of injected cells that migrate to the injury site was significantly lower compared to the local injection [107], and further studies are needed to address the contributing mechanisms to the improved bone repair by the MSC’s systemic administration. Interestingly, in a rat model of open femur fracture, Xu L et al. have shown an improved bone healing effect of systemically administrated Sox11-modified MSCs [108]. The authors show that Sox11 activates the BMP/Smad signaling pathway and Runx2, CXCR4 expression, probably resulting in a synergistic effect that contributes to the effect of Sox11 on the observed MSC-induced enhanced bone repair [108].

In most of these studies, the animal model is subjected to an isolated fracture, in contrast to a generalized bone disorder. However, it is not surprising that the systemic transplantation of MSCs has already been tested in a clinical setting for skeletal disorders. For instance, Horwitz et al. [109] treated osteogenesis imperfecta patients with a systemic transplantation of un-manipulated bone marrow, containing MSCs, resulting in a marked increase in longitudinal growth and bone mineralization, indicating the osteogenic potential of systemically delivered MSCs in the treatment of osteogenesis imperfecta and, eventually other mesenchymal stem cell disorders as well.

For an appropriate systemic delivery of MSCs, it is important to consider the administration route. Intravascular injection of MSCs is the most popular option, as it potentially distributes the cells throughout the entire body. Nonetheless, Castelo-Branco et al. have reported significant entrapment of cells after passing through the lungs and formation of vascular microemboli [110]. In alternative, MSCs can be systemically delivered via intra-arterial injection circumventing the lungs, although embolism was also reported in this delivery route for BM-MSCs in the rat [111].

## 4. Use of MSC-Secretome for Repair of Long Bone Fractures

### 4.1. Advantages and Limitations

The therapeutic potential of MSCs is known to be dependent on the microenvironment that MSCs encounter upon transplantation. These cells have a mode of action that involves a strong paracrine component, since they are able to secrete high levels of growth factors and cytokines. Due to this, the MSCs’ secretome is currently being explored for several clinical contexts, either using conditioned media or EV. The use of MSCs secretome can bring several advantages for the field of tissue engineering and regenerative medicine, since it can overcome the high capital investment on cell therapies, the expensive cell culture techniques and the complicated safety and quality management issues [112].

Still, MSCs heterogeneity regarding their source, donor, protocol for cell isolation and expansion has impaired the full comprehension of the role of MSCs preconditioning on their therapeutic role [113]. The advances in high-throughput techniques and bioinformatic tools, together with the widespread research on EVs/exosomes, as well as the less strict regulations of cell-free approaches in regenerative medicine are expected to boost the clinical use of MSCs secretome in the future.

However, the current number of studies investigating MSC conditioned media for bone regeneration, using in vivo models, either in critical-size defects or not, is still very limited. Conversely, the MSCs secretome presents interesting molecules, which can serve as promoters of bone repair. Considering that it could be interesting for future studies to genetically modulate MSCs in order to increase production of pro-regenerative secreted proteins, we detail below the most promising molecules and review the in vivo and clinical studies that used the MSCs secretome (from non-genetically modified MSCs) to induce bone repair.

### 4.2. Protein Content of MSC-Secretome

The MSCs secretome is a cocktail of growth factors including adhesion factors (e.g., VCAM-1, ICAM-1), antioxidant factors (e.g., catalase), anti-apoptotic factors (e.g., TRAIL), regulators of cell proliferation and differentiation (e.g., EGF), chemoattractors (e.g., MCP-1, RANTES, etc.), immunoregulators (e.g., TGF-β, TSG-6, IDO), proteinases (e.g., MMPs), neuroprotectors (BDNF), osteogenic factors (BMP), angiogenic factors (e.g., VEGF, angiogenin, etc.) [113]. The angiogenic potential of MSCs secretome has been widely known for a long time, and it has been attributed to Cyr61 polypeptide [114], among other proteins [29].

Nevertheless, the composition of the MSCs’ secretome is not constant but instead can be modulated by preconditioning MSCs during in vitro culture. This pre-conditioning period can make use of a 3D microenvironment, pharmacological compounds, inflammatory cytokines and/or hypoxia, and it has been recently reviewed by our group [113]. For example, hypoxia pre-conditioning of ASCs enriched their secretome in 43 angiogenic factors, with 11 of them reported as being involved in bone regeneration (IGF-1, TGF-β1, VEGF, Angiogenin, IL- 6, PDGF-BB, FGFb, EGF, RANTES, MCP-1, and MCP3) [115]. Similar growth factors have been found in bone marrow-derived MSCs. But ASCs demonstrate several advantages over BM-derived MSCs, besides being obtained by a less invasive harvesting procedure, and higher numbers of stem cell progenitors could be isolated, Dufrane et al. also reported that VEGF produced by ASCs is not affected by donor’s age, contrarily to BM-MSCs [116].

Pro-inflammatory cytokines, normally found in injured tissues where MSCs are transplanted, have also a strong impact on MSCs secretome. Particularly, a pro-inflammatory microenvironment is known to enhance tissue inhibitor of metalloproteinase-1 (TIMP1), a key effector molecule responsible for the anti-angiogenic properties MSC within an inflammatory microenvironment [117]. The pre-conditioning can be efficient in short-time periods, such as 2 h. Czekanska et al. showed that a 2 h stimulation of MSCs with IL-1β is enough to increase the secretion of a wide range of proteins with chemotactic, proinflammatory and angiogenic properties including the G-CSF, SDF-1 and SCF [118]. Priming of umbilical cord tissue-derived MSCs in 3D culture was shown to lead to a significantly higher therapeutic potential of these cells’ secretome in an adjuvant-induced model for arthritis, increasing their content in anti-inflammatory cytokines such as IL-10 and LIF, along with trophic factors involved in tissue regeneration, such as PDGF-BB, FGF-2, I-309, SCF, and GM-CSF [119].

### 4.3. Animal Models/Clinical Testing Using MSC Secretome

Some years ago, Dr. Ueda Lab has started to explore the effect of stem-cell-cultured conditioned media on bone regeneration. The authors reported for the first time that cultured conditioned media from human BM–MSCs enhanced the migration, proliferation and expression of osteogenic marker genes, such as osteocalcin and Runx2 of endogenous MSCs, mostly due to increased content of cytokines such as IGF-1 and VEGF [120]. Moreover, in a rat calvaria bone defect model, implantation of agarose scaffolds with MSCs secretome induced a greater area of newly regenerated bone, almost covering the defect upon 8 weeks after implantation, even higher that a group of animals that received MSCs [120]. This work opened new perspectives for the therapeutic use of the MSCs secretome in bone repair/regeneration that has shown beneficial effects in different models such as: (1) Bone defect in rat calvaria [112]; (2) rat periodontal bone defect using collagen sponges embedded in MSCs secretome [121]; (3) rat model of bisphosphonate-related osteonecrosis of the jaw, meaning the exposure of necrotic bone in the oral cavity, in which MSCs secretome has shown enhancement of new bone formation and osteoclasts [122]; (4) rabbit maxillary sinus cavities, in which β-TCP scaffolds soaked with MSCs secretome was grafted, increasing the migration and proliferation of endogenous MSCs and promoting early bone regeneration in rabbit sinus, after maxillary sinus floor elevation [123]; and (5) dog periodontal bone defect, using commercially available collagen-based sponges (TERUPLUG) soaked with MSCs secretome [124]. More recently, a role of MSCs secretome on osteoclast differentiation and expression of master regulatory transcriptional factors for osteoclastogenesis has been reported [125].

The beneficial effects of MSCs secretome for bone repair/regeneration were in great part attributed to its enrichment in angiogenic factors, such as IGF-1, VEGF, and TGF-β1. The angiogenic potential of MSCs secretome was confirmed by an increase of HUVECs tube formation blood vessel formation and cell migration, that was impaired when blocking VEGF [126]. In addition, when a cytokine cocktail with the same angiogenic factors in similar concentrations to those found in MSCs secretome was tested, similar results were obtained concerning enhancement of cell migration, tube formation and expression of osteogenic and angiogenic genes and increase of bone regeneration *in vivo*. Nevertheless, when IGF-1, VEGF, and TGF-β1 were depleted from MSCs secretome, the in vitro effects were decreased but in vivo bone regeneration was not impaired [127].

The promising in vivo results obtained so far led to the use of MSCs secretome in clinical trials for bone repair/regeneration. The first human study conducted used conditioned media from commercially available BM-derived MSCs that was administered to 8 patients suffering from severe alveolar bone atrophy and needing bone augmentation. The patients received either β-TCP or shell-shaped atelocollagen sponge (ACS) scaffold grafts soaked in MSCs secretome. Minor inflammation of the local tissues was observed with less infiltration of inflammatory cells recorded. The scaffold was gradually replaced by newly formed bone, with no records of bone resorption in any of the cases and early mineralization observed in the augmented bone [128]. Another study was conducted to evaluate the safety of use of the secretome of BM-MSC for maxillary sinus floor elevation (SFE). In this study with 6 patients, the secretome of BM-MSCs was mixed with porous β-TCP scaffolds and histological analysis revealed a significant difference in newly formed bone area between the groups. In particular, bone volume in the center of the augmented area was significantly greater in patients that were implanted with β-TCP scaffolds embedded with MSCs secretome, revealing that this strategy is safe and has a great osteogenic potential for bone regeneration [129].

## 5. Potential of MSCs Therapies for Non-Union Bone Defects in the Clinic

In clinical settings, MSCs have been used to treat non-union bone defects, particularly when other therapeutic approaches fail. The potential of undifferentiated cells to increase chances of metastasis (for instance when repairing bone after tumor resection), and to undergo uncontrolled proliferation were reported as significantly reduced by promoting osteogenic differentiation [130]. Autologous transplantation of adipose-derived MSCs for treatment of bone non-union was reported as feasible and safe in patients that were not responding to conventional therapies [131]. A clinical study comparing the injection of bone marrow aspirate, which contains osteogenic precursors, in diabetic and non-diabetic patients found similar cellularity and MSC numbers, and a recovery of 76% of diabetic tibial non-unions, compared with 91% in non-diabetic patients [132]. Another study using BM-MSC for ankle non-unions of diabetic patients reported decreased complications and increased bone healing [133]. A case reported the success of allogenic UC-MSC in a hydroxyapatite scaffold and combined with BMP-2 to repair an infected femoral non-union fracture, compared to the previous 4 surgeries done to the patient, including autologous bone grafting [134]. In a pilot study, patients with large diaphyseal defects treated with HA ceramic scaffold seeded with autologous BM-MSCs presented a complete fusion between the implant and host bone, in critical-size long bone defects [71].

In a recent clinical study, MSCs directly sorted from the bone marrow of the patients, seeded on β-TCP, and transplanted into the patient without in vitro culture performed as well as bone [135]. Another open label clinical study showed that autologous intraosseous MSC injection, improved the outcome of the external fixation Ilizarov method for tibial non-union [136]. A multicenter open, comparative, three-arm randomized clinical trial to compare the efficacy, at 1 and 2 years, of autologous human BM-MSCs, expanded treatment, versus iliac crest autograft, to enhance healing in non-union fractures has been set up [137]. Recent results of a European multicentric first in human clinical trial, demonstrated the safety and feasibility of combining commercially available biphasic calcium phosphate bioceramic granules and autologous MSCs expanded from BM under good manufacturing practices in patients with long bone pseudoarthrosis and tibia or humerus non-unions, showing that 26 out of 28 patients were radiologically healed at 1 year [138].

Contrary to MSC alone, the use of genetically modified MSC in clinical trials remains changeling. Although molecular engineered-MSC show improvement of bone regeneration in pre-clinical studies, its translation into the clinics will only be effective after ensuring safety and minimizing the risks, which can be achieved by optimizing methodologies and improving targeting effectiveness.

## 6. Conclusions and Future Prospects: The Advantages and Limitations of Using MSC in Bone Regeneration

The use of MSCs to promote bone repair has shown promising results and may help to overcome current limitation of bone grafts (autographs, allographs or commercial bone grafts) or alternative biomaterial implants. These cells are easy to collect and expand in vitro and present ideal properties for the treatment of difficult to heal bone defects, mainly due to their immunomodulatory and osteogenic differentiation capacities. However, there are still several factors that may be limiting their use and increasing variability between studies, including the MSCs source and the time and number of MSCs to implant. There is also the need to fully characterize the MSCs population and evaluate the best approach depending on the type and location of the bone trauma/defect. MSC grafting in patients with cancer history should be carefully considered and conflicting results in the literature about the role of MSC in cancer should be taken into account, as both tumor suppressive and tumor progressive/pro-metastatic functions have been reported [139]. This is particularly relevant in large bone defects created after surgery for tumor resection. In these cases, if an intervention with engineered-MSC is considered, the molecules used to promote bone regeneration/repair should also induce an anti-proliferation, anti-metastatic and pro-apoptotic role in cancer cells.

Furthermore, when MSC in vitro expansion is needed, either to increase the number of cells to be transplanted into the defect size, or for gene-delivery purposes, it also generates some limitations. The MSC expansion process is still not fully standardized and automated, and variability depends on the number of passages, type of media and the use of serum (lot-to-lot variations, use of animal-free media or presence of xenogenic proteins, etc.), or risk of contaminants. So far, the pre-clinical results using MSC in in vivo models showed enhanced of bone regeneration. Importantly, MSCs may be genetically engineered to enhance their proliferative capacity, osteogenic differentiation potential, immunomodulatory potential or to modulate their secretome. Similar to the limitations in other genetically modified cells, genetic modulation of MSCs still presents safely issues, particularly when viral vectors are used. However, as molecular biology techniques become more refined, the potential for using engineered MSCs, or their secretome, in clinical practice for the treatment of bone defects increases.
